# Recent advances in the molecular mechanisms determining tissue sensitivity to glucocorticoids: novel mutations, circadian rhythm and ligand-induced repression of the human glucocorticoid receptor

**DOI:** 10.1186/1472-6823-14-71

**Published:** 2014-08-25

**Authors:** Nicolas C Nicolaides, Evangelia Charmandari, George P Chrousos, Tomoshige Kino

**Affiliations:** 1 Division of Endocrinology, Metabolism and Diabetes, First Department of Pediatrics, University of Athens Medical School, ‘Aghia Sophia’ Children’s Hospital, Athens 11527, Greece; 2 Division of Endocrinology and Metabolism, Clinical Research Center, Biomedical Research Foundation of the Academy of Athens, Athens 11527, Greece; 3 Saudi Diabetes Study Research Group, King Fahd Medical Research Center, King Abdulaziz University, Jeddah, Saudi Arabia; 4 Unit on Molecular Hormone Action, Program in Reproductive and Adult Endocrinology, Eunice Kennedy Shriver National Institute of Child Health and Human Development, National Institutes of Health, Bethesda, Maryland 2089, USA

**Keywords:** Glucocorticoid receptor, Glucocorticoid resistance, Glucocorticoid hypersensitivity, Glucocorticoid signal transduction

## Abstract

Glucocorticoids are pleiotropic hormones, which are involved in almost every cellular, molecular and physiologic network of the organism, and regulate a broad spectrum of physiologic functions essential for life. The cellular response to glucocorticoids displays profound variability both in magnitude and in specificity of action. Tissue sensitivity to glucocorticoids differs among individuals, within tissues of the same individual and within the same cell. The actions of glucocorticoids are mediated by the glucocorticoid receptor, a ubiquitously expressed intracellular, ligand-dependent transcription factor. Multiple mechanisms, such as pre-receptor ligand metabolism, receptor isoform expression, and receptor-, tissue-, and cell type-specific factors, exist to generate diversity as well as specificity in the response to glucocorticoids. Alterations in the molecular mechanisms of glucocorticoid receptor action impair glucocorticoid signal transduction and alter tissue sensitivity to glucocorticoids. This review summarizes the recent advances in our understanding of the molecular mechanisms determining tissue sensitivity to glucocorticoids with particular emphasis on novel mutations and new information on the circadian rhythm and ligand-induced repression of the glucocorticoid receptor.

## Background

In humans, glucocorticoids are synthesized by the adrenal cortex and released following activation of the hypothalamic-pituitary-adrenal (HPA) axis, and play an important role in the maintenance of resting and stress-related homeostasis. Named for their effects on glucose metabolism, glucocorticoids are involved in almost every cellular, molecular and physiologic network of the organism, and regulate a broad spectrum of physiologic functions essential for life, including growth, reproduction, cognition, behavior, cell proliferation and survival, as well as immune, central nervous system (CNS) and cardiovascular functions [1-3]. Given their powerful anti-inflammatory and immunosuppressive actions, synthetic glucocorticoids represent one of the most widely used therapeutic compounds employed in the treatment of acute and chronic inflammatory/autoimmune and lymphoproliferative disorders [1,3]. However, chronic exposure to glucocorticoids in patients with such disorders leads to multiple adverse effects. Sometimes glucocorticoid resistance of the affected organ or tissue may develop, representing a major challenge for the treatment of these conditions [4].

At the cellular level, the actions of glucocorticoids are mediated by the human glucocorticoid receptor (*hGR*, *NR3C1*), which belongs to the steroid/thyroid/retinoic acid nuclear receptor superfamily of transcription factors [2,5]. Consistent with the pleiotropic effects of glucocorticoids, the hGR is ubiquitously expressed in all human tissues and cells, and is necessary for life after birth [6]. The hGR protein is encoded by exons 2–9 of the *hGR* gene (located on chromosome 5) and is composed of four distinct regions: the amino-terminal domain (NTD), the DNA-binding domain (DBD), the hinge region and the ligand-binding domain (LBD) [2,5]. Alternative splicing of hGR precursor mRNA gives rise to 5 hGR protein subtypes that have been termed hGRα, hGRβ, hGRγ, hGR-A and hGR-P. An additional cohort of eight receptor proteins (hGRα-A, hGRα-B, hGRα-C1, hGRα-C2, hGRα-C3, hGRα-D1, hGRα-D2, and hGRα-D3) is produced by alternative translation initiation from hGR mRNA [2,5,7]. hGRα-A is the classic full-length 777-amino acid receptor that is generated from the first translation initiation codon [2,5,7]. The other hGRα isoforms have progressively shorter NTDs, possess both common and unique properties and may differentially transduce the glucocorticoid signal to target tissues depending on their selective relative expression and inherent activities. All translational isoforms have similar affinity for the ligand and ability to bind to DNA, consistent with the presence of a common LBD. However, they display differences in their subcellular distribution, with hGRα-D residing constitutively in the nucleus of cells, a fact that indicates that sequences in the NTD of the hGR may play an important role in nuclear translocation, nuclear export and/or cytoplasmic retention of the receptor. In addition, these translational isoforms display significant differences in their ability to regulate gene expression, with the hGRα-C isoforms being the most active, and the hGRα-D subtypes being the most “deficient” in their ability to transactivate glucocorticoid-responsive genes [7]. Recent evidence suggests that hGRα-C3 displays higher transcriptional activity than the other hGRα isoforms, owing to increased recruitment of coactivators at the promoter regions of target genes [8]. In addition to the hGR isoforms generated by alternative splicing or alternative initiation of translation, four novel receptor variants with multiple amino acid replacements/truncation have been described [hGR NS-1, hGR DL-1, hGR-S1 and hGR-S1 (-349A)] [9,10]. Their functional role remains to be elucidated.

The hGR regulates gene expression by either transcriptional activation (transactivation) or transcriptional repression (transrepression). Prior to binding to glucocorticoids, the hGR resides mostly in the cytoplasm of cells as part of a large multiprotein complex [2,11]. Upon ligand-induced activation, the receptor undergoes conformational changes that result in dissociation from this multiprotein complex and translocation into the nucleus, where it binds to glucocorticoid-response elements (GREs) in the promoter region of target genes [2,11,12]. The latter contain hexamer domains in an inverted palindrome arrangement separated by 3 base pairs in the regulatory regions of target genes and regulate their expression positively or negatively through interaction with coactivators [12] or corepressors [2,11], respectively. Glucocorticoids may mediate anti-inflammatory effects via direct binding of hGR to evolutionarily conserved negative GREs (nGREs), which contain an inverted tetrameric palindrome separated by 0–2 base pairs that is distinct from the classic GREs [13]. The ligand-activated hGR can also modulate gene expression independently of DNA-binding, by interacting with other transcription factors, such as nuclear factor-κB (NF-κB), activator protein-1 (AP-1), p53 and signal transducers and activators of transcription (STATs). The interaction of hGR with the pro-inflammatory transcription factors NF-κB and AP-1 inhibits their activity and accounts for the major anti-inflammatory and immunosuppressive effects of glucocorticoids [3]. Although the transcriptional activity of hGR is primarily governed by ligand binding, post-translational modifications also play important roles. These covalent changes include methylation, acetylation, nitrosylation, sumoylation, ubiquitination and phosphorylation, and may affect receptor stability, subcellular localization, as well as the interaction of hGR with other proteins [11].

In addition to the above-described genomic actions, glucocorticoids can induce some effects via the GR within seconds or minutes. The non-genomic rapid glucocorticoid actions appear to be mediated by membrane bound GRs, which trigger the activation of kinase signaling pathways, thus influencing many CNS and other tissue functions [14]. On the other hand, the MR functions as the 2nd glucocorticoid receptor in some tissues (e.g. limbic structure of the brain and adipose tissue), which do not express the glucocorticoid-inactivating 11β-hydroxysteroid dehydrogenase 2, and cooperates with the classic GR to regulate expression of common and/or distinct target genes [15].

The cellular response to glucocorticoids displays profound variability both in magnitude and in specificity of action [2,11]. Multiple mechanisms exist to generate diversity, as well as specificity in the response to glucocorticoids, such as pre-receptor ligand metabolism, receptor isoform expression, and receptor-, tissue-, and cell type-specific factors. Furthermore, recent findings from *in vitro* and *in vivo* studies have demonstrated the important new role of old molecules, such as the serum- and glucocorticoid-inducible kinase 1 (SGK1) [16,17] and FK506 -binding protein 51 (FKBP5) [18,19], in tissue sensitivity to glucocorticoids and associated pathologic conditions. In addition to protein-protein interactions, tissue responsiveness to glucocorticoids has become more complicated since the identification and functional characterization of *hGR* polymorphisms [20-24]. Interestingly, MR polymorphisms may also play some roles in tissue glucocorticoid sensitivity [25]. Any of the above-described molecular mechanisms may lead to alterations in tissue sensitivity to glucocorticoids, which may take the form of *glucocorticoid resistance* or *glucocorticoid hypersensitivity* and may be associated with significant morbidity (Table 1) [26]. This review summarizes the recent advances in the molecular mechanisms underlying tissue sensitivity to glucocorticoids, with particular emphasis on novel mutations and new information on the circadian rhythm of tissue sensitivity to glucocorticoids, and ligand-induced repression of the glucocorticoid receptor.

**Table 1:** Expected clinical manifestations in tissue-specific glucocorticoid resistance or hypersensitivity

**Target tissue**	**Glucocorticoid hypersensitivity = Glucocorticoid excess**	**Glucocorticoid resistance = Glucocorticoid deficiency**
Central nervous system	Insomnia, anxiety, depression, defective cognition	Fatigue, somnolence, malaise, defective cognition
Liver	+ Gluconeogenesis, + lipogenesis	Hypoglycemia, resistance to diabetes mellitus
Fat	Accumulation of visceral fat (metabolic syndrome)	Loss of weight, resistance to weight gain
Blood vessels	Hypertension	Hypotension
Bone	Stunted growth, osteoporosis	
Inflammation/immunity	Immune suppression, anti-inflammation, vulnerability to certain infections and tumors	+ Inflammation, + autoimmunity, + allergy

Modified from Reference [30].

### Pathologic natural *hGR* gene mutations causing primary generalized glucocorticoid resistance or Chrousos syndrome

**Figure 1:**
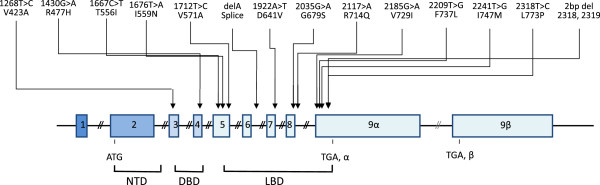
**Schematic representation of the known mutations of the *hGR *gene causing Chrousos syndrome.** DBD: DNA-binding domain; LBD: ligand-binding domain; NTD: amino terminal domain.

Natural *hGR* gene mutations impair the molecular mechanisms of hGR action and alter tissue sensitivity to glucocorticoids [27-49]. The majority of the natural *hGR* mutations described to date are associated with Chrousos syndrome, a rare, familial or sporadic genetic condition characterized by generalized, partial, target-tissue insensitivity to glucocorticoids (Figure 1). Affected subjects have compensatory activation of the HPA axis and elevations in circulating cortisol and adrenocorticotropic hormone (ACTH) concentrations throughout the 24-hour period without, however, any clinical manifestations of hypercortisolism. The excess ACTH secretion results in adrenal hyperplasia, and increased production of adrenal steroids with mineralocorticoid activity [cortisol, deoxycorticosterone (DOC) and corticosterone] and/or androgenic activity [androstenedione, dehydroepiandrosterone (DHEA) and DHEA-sulfate (DHEAS)], and the corresponding clinical phenotype (Tables 2 and 3) [27-45,47-49].

**Table 2:** Clinical manifestations and diagnostic evaluation of primary generalized glucocorticoid resistance or Chrousos Syndrome

**Clinical presentation**	**Diagnostic evaluation**
Apparently normal glucocorticoid function	Absence of clinical features of Cushing syndrome
Asymptomatic	Normal or elevated plasma ACTH concentrations
Hypoglycemia	Elevated plasma cortisol concentrations
Chronic fatigue (glucocorticoid deficiency?)	Increased 24-hour urinary free cortisol excretion
Mineralocorticoid excess	Normal circadian and stress-induced pattern of cortisol and ACTH secretion
Hypertension	Resistance of the HPA axis to dexamethasone suppression
Hypokalemic alkalosis	Thymidine incorporation assays: Increased resistance to dexamethasone-induced suppression of phytohemaglutinin-stimulated thymidine incorporation compared to control subjects
Androgen excess	Dexamethasone-bindings assays: Decreased affinity of the glucocorticoid receptor for the ligand compared to control subjects
Children: Ambiguous genitalia at birth*, premature adrenarche, precocious puberty	Molecular studies: Mutations/deletions of the glucocorticoid receptor
Females: Acne, hirsutism, male-pattern hair loss, menstrual irregularities, oligo-anovulation, infertility	
Males: Acne, hirsutism, oligospermia, adrenal rests in the testes, infertility	
Increased HPA axis activity (CRH/ACTH hypersecretion)	
Anxiety	
Adrenal rests	

Modified from References [28] and [29].

*This is the only case of ambiguous genitalia documented in a child with 46,XX karyotype who also harbored a heterozygous mutation of the 21-hydroxylase gene.

**Table 3:** Mutations of the human glucocorticoid receptor gene causing primary generalized glucocorticoid resistance or hypersensitivity

**Mutation position**					
**Author (Reference)**	**cDNA**	**Amino acid**	**Molecular mechanisms**	**Genotype**	**Phenotype**
Chrousos *et al.*[27]	1922 (A → T)	641 (D → V)	Transactivation ↓	Homozygous	Hypertension
Hurley *et al.*[32]			Affinity for ligand ↓ (× 3)		Hypokalemic alkalosis
			Nuclear translocation: 22 min		
			Abnormal interaction with GRIP1		
Karl *et al*. [33]	4 bp deletion in		hGRα number: 50% of control	Heterozygous	Hirsutism
	exon-intron 6		Inactivation of the affected allele		Male-pattern hair-loss
					Menstrual irregularities
Malchoff *et al.*[34]	2185 (G → A)	729 (V → I)	Transactivation ↓	Homozygous	Precocious puberty
			Affinity for ligand ↓ (× 2)		Hyperandrogenism
			Nuclear translocation: 120 min		
			Abnormal interaction with GRIP1		
Karl *et al.*[31]	1676 (T → A)	559 (I → N)	Transactivation ↓	Heterozygous	Hypertension
Kino *et al.*[35]			Decrease in hGR binding sites		Oligospermia
			Transdominance (+)		Infertility
			Nuclear translocation: 180 min		
			Abnormal interaction with GRIP1		
Ruiz *et al.*[36]	1430 (G → A)	477 (R → H)	Transactivation ↓	Heterozygous	Hirsutism
Charmandari *et al.*[41]			No DNA binding		Fatigue
			Nuclear translocation: 20 min		Hypertension
Ruiz *et al.*[36]	2035 (G → A)	679 (G → S)	Transactivation ↓	Heterozygous	Hirsutism
Charmandari *et al.*[41]			Affinity for ligand ↓ (× 2)		Fatigue
			Nuclear translocation: 30 min		Hypertension
			Abnormal interaction with GRIP1		
Mendonca *et al.*[37]	1712 (T → C)	571 (V → A)	Transactivation ↓	Homozygous	Ambiguous genitalia
			Affinity for ligand ↓ (× 6)		Hypertension
			Nuclear translocation: 25 min		Hypokalemia
			Abnormal interaction with GRIP1		Hyperandrogenism
Vottero *et al.*[38]	2241 (T → G)	747 (I → M)	Transactivation ↓	Heterozygous	Cystic acne
			Transdominance (+)		Hirsutism
			Affinity for ligand ↓ (× 2)		Oligo-amenorrhea
			Nuclear translocation ↓		
			Abnormal interaction with GRIP1		
Charmandari *et al.*[40]	2318 (T → C)	773 (L → P)	Transactivation ↓	Heterozygous	Fatigue
			Transdominance (+)		Anxiety
			Affinity for ligand ↓ (× 2.6)		Acne
			Nuclear translocation: 30 min		Hirsutism
			Abnormal interaction with GRIP1		Hypertension
Charmandari *et al.*[42]	2209 (T → C)	737 (F → L)	Transactivation ↓	Heterozygous	Hypertension
			Transdominance (time-dependent) (+)		Hypokalemia
			Affinity for ligand ↓ (× 1.5)		
			Nuclear translocation: 180 min		
McMahon *et al.*[43]	2 bp deletion	773	Transactivation ↓	Homozygous	Hypoglycemia
	at nt 2318-9		Affinity for ligand: absent		Fatigability with feeding
			No suppression of IL-6		Hypertension
Nader *et al.*[44]	2141 (G → A)	714 (R → Q)	Transactivation ↓	Heterozygous	Hypoglycemia
			Transdominance (+)		Hypokalemia
			Affinity for ligand ↓ (× 2)		Hypertension
			Nuclear translocation ↓		Mild clitoromegaly
			Abnormal interaction with GRIP1		Advanced bone age
					Precocious pubarche
Zhu Hui-juan *et al.*[45]	1667 (G → T)	556 (T → I)	Not studied yet	Heterozygous	Adrenal incidentaloma
Charmandari *et al.*[46]	1201 (G → C)	401 (D → H)	Transactivation ↑	Heterozygous	Visceral obesity
			Transdominance (+)		Hypercholesterolemia
			Affinity for ligand: N		Hypertriglyceridemia
			Nuclear translocation: N		Hypertension
			Interaction with GRIP1: N		Diabetes type 2
Roberts *et al.*[47]	1268 (T → C)	423 (V → A)	Transactivation ↓	Heterozygous	Fatigue
			Affinity for ligand: N		Anxiety
			No DNA binding		Hypertension
			Nuclear translocation: 35 min		
			Interaction with GRIP1: N		
Nicolaides *et al.*[48]	1724 (T → G)	575 (V → G)	Transactivation ↓	Heterozygous	Melanoma
			Transrepression ↑		Asymptomatic daughters
			Affinity for ligand ↓ (× 2)		
			Nuclear translocation ↓		
			Abnormal interaction with GRIP1		

Modified from References [29] and [30].

Numbers in the parentheses following authors’ names indicate the corresponding references.

**Figure 2:**
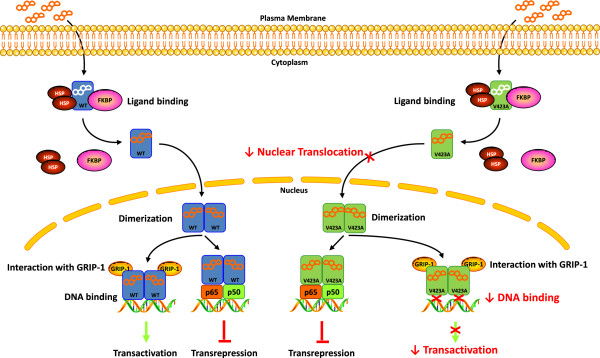
**Μolecular mechanisms through which the natural mutant receptor hGRαV423A causes Chrousos syndrome.** Both the wild-type hGRα and the mutant hGRαV423A reside in the cytoplasm in the absence of ligand by forming a heterocomplex with heat shock proteins (HSP) and FKBP51 (FKBP). Upon binding to ligand, the wild-type hGRα dissociates from the heterocomplex partners and translocates into the nucleus, while this process of the mutant hGRαV423A is significantly delayed. The wild-type hGRα stimulates or represses the transcriptional activity of glucocorticoid-responsive genes by attracting to GRE DNA several coactivators including the glucocorticoid receptor-interacting protein 1 (GRIP-1), or by interacting with other transcription factors, such as NF-κB and activator protein-1 (AP-1). The hGRαV423A demonstrates reduced transactivation activity due to decreased ability to interact with GREs, while its activity to repress the transcriptional activity of other transcription factors is preserved. The hGRαV423A does not exert a dominant negative effect upon the wild type-induced transactivation of glucocorticoid-responsive genes. WT: wild-type human glucocorticoid receptor; V423A: human glucocorticoid receptor V423A; HSP: heat shock proteins; FKBP: immunophilins; GRIP-1: glucocorticoid receptor-interacting protein 1; p65: transcription factor p65; p50: transcription factor p50.

We have recently described a new case of Chrousos syndrome caused by a novel, heterozygous, point mutation (V423A) in the DBD of the hGRα, and investigated the molecular mechanisms through which the hGRαV423A affects glucocorticoid signal transduction. Compared with the wild-type receptor, the mutant receptor hGRαV423A demonstrated a significant reduction (72%) in its ability to transactivate glucocorticoid-responsive genes, impaired ability to bind to GREs and a marked delay (2.6-fold) in the time required to translocate into the nucleus. However, the hGRαV423A did not exert a dominant negative effect upon the wild-type hGRα, had a similar affinity for the ligand, and displayed a normal interaction with the glucocorticoid receptor-interacting protein-1 (GRIP1) coactivator (Figure 2) [47].

Structural biology studies using computer-based creation of the 3-dimentional structure of the hGR DBD harboring the V423A mutation showed that the V423A substitution alters the hydrophobic nature of the first zinc finger of the hGRα DBD, and permits water to diffuse into the nearby zinc-binding site. The hydrophobic valine (V) at position 423 in the hGRα DBD shields the four zinc-binding cysteines (C421, C424, C438, and C441) from water, thereby creating a hydrophobic environment. Replacement of valine (V) by alanine (A) at this position destroys the hydrophobic environment and permits water to diffuse into the ion-binding region of the protein, where it is captured by hydrogen bonds to C424 and C441, and reduces their affinity for binding to GREs [47]. These findings expand our knowledge on the previously described molecular mechanisms of action of natural *hGR* mutants, and may explain the differences observed in the clinical phenotype of subjects with Chrousos syndrome [27-45,47-49].

### CLOCK-mediated acetylation of hGR: Implications for circadian rhythm-mediated regulation of glucocorticoid action in target tissues

In humans, circulating cortisol concentrations are tightly regulated by the central components of the HPA axis and fluctuate naturally in an ultradian and circadian fashion, reaching their zenith in the early morning and their nadir in the late evening [1]. Stavrera *et al*. showed that ultradian glucocorticoid pulses resulted in dynamic association and dissociation of GR with the promoters of target genes leading to cyclic GR-mediated transcriptional regulation [50]. Interestingly, this coupling was not observed upon exposure of cells to synthetic glucocorticoids, since these were shown to prevent the dissociation of GR from the target promoter [50]. In addition to the above findings at the level of target tissues, recent studies have investigated the molecular mechanisms underlying glucocorticoid oscillations [51-53]. Walker *et al.* employed a mathematical model to study how the HPA axis supports ultradian glucocorticoid fluctuations [51]. They demonstrated that the pituitary-adrenal axis can produce ACTH and glucocorticoid ultradian oscillations in the presence of constant levels of CRH [51]. Their results were further confirmed by *in vivo* studies [53]. Indeed, a constant CRH input in the pituitary-adrenal axis resulted in physiological ultradian fluctuations of ACTH and glucocorticoids, whereas higher levels of CRH led to the disruption of the oscillating patterns [53]. On the other hand, the circadian secretion of cortisol is generated by an evolutionary conserved molecular ‘clock’, the circadian clock system, which consists of central and peripheral components [54,55]. The central clock system, located in the suprachiasmatic nucleus (SCN) of the hypothalamus, acts as the ‘master’ oscillator and generator of the body’s circadian rhythm under the strong influence of the light/dark input from the eyes [50,54,55]. Interestingly, the neurons of SCN do not express GR; therefore glucocorticoid feedback does not directly affect the activity of the master SCN clock. The peripheral clock system, which is distributed in all organs and tissues, including the central nervous system outside the SCN, acts generally as a ‘slave’ clock under the regulation of the central SCN clock, by as yet unknown mechanism(s) [54-56]. Both clocks communicate with each other and generate circadian rhythmicity by the coordinated activation/inactivation of self-oscillating transcription factors. Central among them are the circadian locomotor output cycle kaput (CLOCK) and its heterodimer partner brain-muscle-arnt-like protein 1 (BMAL1) [54-56].

**Figure 3:**
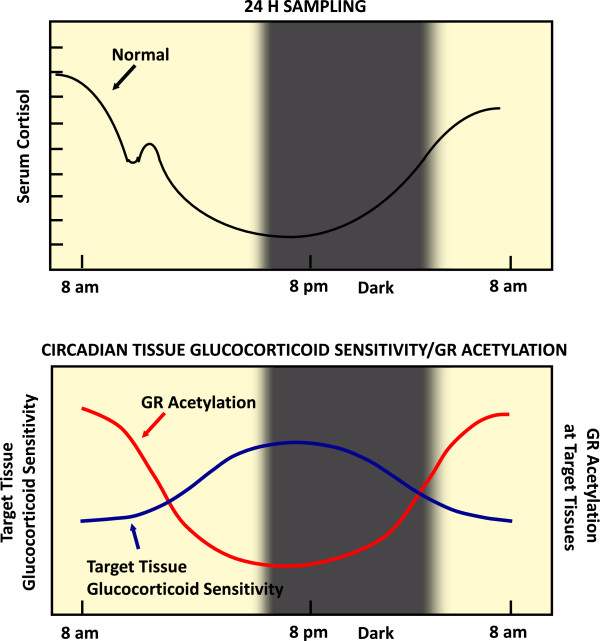
**CLOCK-mediated gene-specific regulation of glucocorticoid action in peripheral target tissues.** Circulating cortisol concentrations in humans fluctuate diurnally, as indicated in the top panel. The expression of glucocorticoid-target genes is also expected to fluctuate depending on the changes of circulating cortisol concentrations. However, this diurnal fluctuation of gene expression is suppressed through acetylation of GR by locally expressed CLOCK/BMAL1 heterodimers, possibly functioning as a local counter-regulatory feedback loop to the circulating glucocorticoids. Thus, high levels of acetylated GR in the morning are associated with low target-tissue sensitivity to glucocorticoids and *vice versa* in the evening and early night. Modified from Reference [56].

We have recently demonstrated that CLOCK physically interacts with the LBD of the hGR and suppresses the hGR-induced transcriptional activity by acetylating multiple lysine residues (480, 492, 494, and 495) in the hinge region of the receptor. This post-translational modification attenuates the binding of hGR to GREs and its ability to influence glucocorticoid-responsive gene expression. Furthermore, the expression of glucocorticoid-responsive genes fluctuated in a circadian fashion, mirroring in reverse phase the *Clock/Bmal1* expression [57]. These findings indicate that CLOCK/BMAL1 is a reverse-phase negative regulator of glucocorticoid action in target tissues, antagonizing the biologic actions of diurnally fluctuating circulating glucocorticoids and providing a local target tissue counter-regulatory feedback loop to the central clock influence on the HPA axis [57]. As a result, tissue sensitivity to glucocorticoids is decreased in the morning (when circulating cortisol concentrations are elevated) and increased in the evening and early night (when cortisol concentrations reach their nadir) (Figure 3).

In addition to these *in vitro* findings, we examined the acetylation status of hGR and the expression of clock-related and glucocorticoid-responsive genes *in vivo* and *ex vivo*, using peripheral blood mononuclear cells from healthy adult volunteers [58]. The levels of acetylated hGR were higher in the morning and lower in the evening, mirroring the fluctuations of circulating cortisol concentrations in reverse phase. All known glucocorticoid-responsive genes tested responded as expected to hydrocortisone, however, some of these genes did not show the expected diurnal mRNA fluctuations *in vivo*. Instead, their mRNA did not oscillate in the absence of endogenous glucocorticoid, indicating that circulating cortisol might prevent circadian GR acetylation-dependent effects in some glucocorticoid-responsive genes *in vivo*. These findings suggest that peripheral CLOCK-mediated circadian acetylation of the hGR functions as a target-tissue, gene-specific counter-regulatory mechanism to the actions of diurnally fluctuating cortisol, effectively decreasing tissue sensitivity to glucocorticoids in the morning and increasing it in the evening and early night [58]. Furthermore, in another recent study that determined the mRNA expression of ~190 hGR action-regulating and glucocorticoid-responsive genes in subcutaneous fat biopsies obtained from 25 obese subjects, circulating cortisol concentrations in the evening were more important in regulating the mRNA expression of glucocorticoid-responsive genes than those in the morning [59]. Therefore, it appears that the higher tissue sensitivity to glucocorticoids in the evening, owing to reduced hGR acetylation by CLOCK, underlies the stronger effect of elevated circulating cortisol concentrations on the expression of glucocorticoid-regulated genes in the evening and early night.

In line with our *in vitro*, *in vivo* and *ex vivo* results, Lamia *et al.* recently showed that CRY1 and CRY2 interacted with the GR in a ligand-dependent fashion, leading to direct inhibition of GR-mediated transactivation of glucocorticoid-responsive genes [60]. Interestingly, this CRY-GR interaction did not affect the transrepressing actions of GR on many inflammatory genes, indicating that cryptochromes, when interact with the GR, could influence the expression of a separate group of GR target genes. This dissociation of the transactivating and transrepressing activities of the GR might reduce the frequency of the undesirable metabolic effects during long-term administration of synthetic glucocorticoids [60]. Moreover, a very recent study demonstrated that CLOCK/BMAL1 reduced both maximal GR transactivation of target genes and the efficacy of the receptor to increase the transcription rate of glucocorticoid-responsive genes. On the other hand, the PER1/CRY1 complex reduced the maximal GR transactivation, but not the efficacy of the receptor, suggesting that both elements of the positive and negative arm of the clock system repress GR transcriptional activity [61].

The circadian clock system and the HPA axis regulate the activity of one another through multilevel interactions to ultimately coordinate homeostasis against the day/night change and various unforeseen random internal and external stressors. Uncoupling of or dysfunction in either system alters internal homeostasis and causes pathologic changes virtually in all organs and tissues, including those responsible for intermediary metabolism and immunity [62]. Disrupted coupling of cortisol secretion and target tissue sensitivity to glucocorticoids may account for i) the development of central obesity and the metabolic syndrome in chronically stressed individuals, whose HPA axis circadian rhythm is characterized by blunting of the evening decreases of circulating glucocorticoids, as a result of enhanced input of higher centers upon the hypothalamic paraventricular nucleus secretion of CRH and AVP; and ii) the increased cardiometabolic risk and increased mortality of rotating shift workers or subjects exposed to frequent jet lag because of traveling across time zones [56,63]. In addition, given that tissue sensitivity to glucocorticoids is increased in the evening, clinicians should avoid the administration of high doses of glucocorticoids for the treatment of adrenal insufficiency or congenital adrenal hyperplasia at night, because they increase the possibility of glucocorticoid-related side effects.

At pharmacological concentrations, the transactivational activity of glucocorticoids is correlated with the side effects of these steroids, while their transrepressive activity is associated mostly with their beneficial anti-inflammatory activity. Since CLOCK may differentially regulate these two major class actions of glucocorticoids, administration of these steroids at a specific period of the circadian cycle might increase their pharmacological efficacy, while at the same time reduce their unwanted side effects [62].

### Ligand-induced down-regulation (repression) of *hGR* gene expression

Glucocorticoids are among the most potent and effective agents for treating inflammatory diseases and hematologic malignancies. However, a number of patients are often resistant to treatment with glucocorticoids [1-4]. Therefore, it is important to determine the molecular mechanisms underlying this phenomenon in order to adequately treat patients with these disorders.

Recent studies investigated the molecular mechanisms responsible for repression of *hGR* gene transcription by glucocorticoids. They demonstrated that glucocorticoid treatment resulted in a rapid decrease in nascent hGR RNA, with maximal repression being sustained for 6 to 8 h. They also identified a functional nGRE in exon 6 of the *hGR* gene, which played an important role in the repression of the *hGR* gene. Upon ligand-induced activation, the hGR bound to the nGRE on exon 6 and recruited NCoR1 (nuclear receptor corepressor) and HDAC3 (histone deacetylase 3) to the promoter-proximal region, thereby forming a repression complex and inhibiting hGR transcription [64]. These findings indicate that the hGR concentration can be down-regulated coordinately with excess ligand, regardless of the combinatorial associations of tissue-specific transcription factors. Therefore, although glucocorticoid-induced down-regulation of hGR represents a mechanism for maintaining glucocorticoid homeostasis in normal cells, it appears to have the potential to limit the therapeutic response to glucocorticoids in inflammatory and malignant conditions. As a result, long-term glucocorticoid treatment in patients with the above disorders may lead to constitutive hGR transrepression and glucocorticoid resistance.

In addition to glucocorticoid-induced down-regulation of hGR, the expression of the receptor has been shown to be influenced by epigenetic modifications which occurred in the context of gene-environment interactions. In their pioneering study, Weaver and collaborators demonstrated that maternal behavior had a pivotal role in stress responses of offspring. Indeed, rats exposed to low levels of pup licking and grooming (LG) and arched-back nursing (ABN) by their mothers, had increased methylation of the 1_7_ promoter of the *GR* gene, leading to decreased levels of GR in the hippocampus and altered response of the HPA axis to stressors [65]. This epigenetic alteration of GR expression could also be involved in drug-induced glucocorticoid resistance. Moreover, GR protein levels were found to be reduced by micro-RNAs (miRs), such as miR-18a [66], -18 and -124a [67] in neuronal tissues. Uchida *et al.* used Fischer 344 (F344) rats, a well-known stress-hyperresponsive model, and showed that this strain, upon a 14-day repeat restrain stress, had increased levels of miR-18a and decreased GR protein expression in the PVN, compared with control Sprague–Dawley (SD) rats [66]. Further studies have supported the implication of microRNAs, specifically miR-18 and -124a, in brain responsiveness to glucocorticoids. Both miR-18 and -124a down-regulated the GR, establishing their critical role in the regulation of glucocorticoid responsiveness of the brain [67]. On the other hand, the specific down-regulation of the GR in newborn hippocampal cells, using viral-mediated RNA-interference *in vivo*, increased their neuronal differentiation and migration, as well as their basal excitability. These morphological and functional alterations of hippocampal cells resulted in impairments in memory consolidation [68]. Therefore, blockade of selective GR-dependent processes in brain would be therapeutically beneficial. Zalachoras *et al*. demonstrated that the non-steroidal GR ligand 1*H*-pyrazolo[3,4-*g*]hexahydroisoquinoline or C108297 selectively modulated GR activity in the brain by suppressing CRH gene expression while enhancing GR-dependent memory consolidation by acting as a unique GR modulator [69]. In contradistinction, C108297 antagonized GR-mediated reduction in neurogenesis in the hippocampus following long-term administration of glucocorticoids [69]. Thus, C108297 has a potential to selectively abrogate pathogenic processes associated with GR in the brain, while retaining some beneficial actions of this receptor.

## Conclusions

The glucocorticoid receptor is a ubiquitously expressed intracellular, ligand-dependent transcription factor, which mediates the action of glucocorticoids and influences physiologic functions essential for life. The traditional view that glucocorticoids exert their diverse effects through one receptor protein has changed dramatically over the last two decades with the discovery of multiple hGR isoforms arising from the single *hGR* gene. hGR subtypes with unique expression and gene regulatory profiles are generated by alternative splicing of the nascent transcript, alternative translation initiation of the mature mRNA, and post-translational modifications of the receptor protein. The capacity of a cell to generate dozens of hGR isoforms that control specific sets of genes and/or differentially regulate common sets provides enormous potential for signaling diversity. Further contributing to the tissue- and cell-specific effects of glucocorticoids is the potential for these isoforms to heterodimerize with each other and cross-talk with other signaling molecules. The combinatorial nature of glucocorticoid signaling pathways indicates that alterations in hGR action may have important implications for many critical biological processes and may account for the alterations in tissue sensitivity to glucocorticoids, as well as the variations in response to glucocorticoid treatment documented in clinical practice. A greater understanding of the role that hGR heterogeneity plays in the cellular response to glucocorticoids should aid in the development of new glucocorticoid compounds with selective activities that may offer the prospect of an improved outcome with lesser side effects.

## Competing interests

The authors declare that they have no competing interests.

## Authors’ contributions

NCN carried out the literature search, participated in the preparation of Tables and Figures, and revised the manuscript. EC carried out the literature search, the analysis of the data and wrote the manuscript. GPC and TK reviewed the manuscript critically and offered their comments. All authors read and approved the final manuscript.

## Pre-publication history

The pre-publication history for this paper can be accessed here:

http://www.biomedcentral.com/1472-6823/14/71/prepub
